# Population pharmacokinetics of tofacitinib in patients with psoriatic arthritis 

**DOI:** 10.5414/CP203516

**Published:** 2019-07-19

**Authors:** Rujia Xie, Chenhui Deng, Qiang Wang, Keith S. Kanik, Timothy Nicholas, Sujatha Menon

**Affiliations:** 1 Pfizer Inc, Shanghai, China, and; 2 Pfizer Inc, Groton, CT, USA

**Keywords:** Janus kinase inhibitor, population pharmacokinetics, psoriatic arthritis, tofacitinib

## Abstract

Objective: Tofacitinib is an oral Janus kinase inhibitor for the treatment of psoriatic arthritis (PsA). This analysis characterized the pharmacokinetics (PK) of tofacitinib in adult patients with active PsA and evaluated the impact of covariates (baseline characteristics) on the disposition of tofacitinib. Materials and methods: Data were pooled from two phase 3 studies of tofacitinib of up to 12 months’ duration in patients with active PsA (OPAL Broaden (NCT01877668); OPAL Beyond (NCT01882439)). This analysis included 650 tofacitinib-treated patients with 3,252 tofacitinib plasma concentration measurements. Tofacitinib PK was described using a one-compartment disposition model parameterized in terms of apparent oral clearance (CL/F) and apparent volume of distribution (V/F) with first-order absorption rate (Ka) and a lag time. Covariates evaluated were baseline age, baseline body weight, sex, race, ethnicity, baseline creatinine clearance (BCCL), and baseline C-reactive protein. Results: The estimates (95% confidence interval) of PK model parameters of a reference patient were CL/F: 20.4 (18.6, 21.8) L/h; V/F: 110 (108, 113) L; and Ka: 13.8 (12.1, 16.6)/h. Among the covariates, only BCCL led to clinically relevant changes in exposure; however, this was consistent with the known contribution of renal excretion to the total clearance of tofacitinib (~ 30%). Conclusion: Tofacitinib did not require dose modification or restrictions for age, body weight, sex, race, ethnicity, or baseline disease severity in patients with active PsA based on the magnitude of exposure relative to a reference patient. Dosing adjustments for renal impairment were derived from a separate phase 1 study.

**What is known about this subject**

- Psoriatic arthritis (PsA) is a chronic, immune-mediated inflammatory arthritis, treatments for which can include conventional synthetic disease-modifying antirheumatic drugs (csDMARDs), targeted synthetic DMARDS, or biologic DMARDs.
- Tofacitinib is an oral Janus kinase inhibitor for the treatment of PsA. The efficacy and safety of tofacitinib 5 and 10 mg twice daily in combination with a csDMARD have been demonstrated in two phase 3 trials of up to 12 months’ duration in patients with active PsA and an inadequate response to csDMARD or tumor necrosis factor inhibitor therapy.
- The pharmacokinetics of tofacitinib are characterized by rapid absorption and elimination, with no evidence of unexpected systemic accumulation.

**What this study adds**

- The impact of covariates (baseline characteristics) on the pharmacokinetics of tofacitinib determined that, in patients with active PsA, tofacitinib did not require dose modification or restrictions for age, body weight, sex, race, ethnicity, or baseline disease severity, based on the magnitude of exposure relative to a reference patient.

## Introduction 

Psoriatic arthritis (PsA) is a chronic, immune-mediated inflammatory arthritis characterized by peripheral joint inflammation and destruction, psoriatic skin lesions, enthesitis, dactylitis, spondylitis, and progressive disability [1]. 

Current pharmacologic treatments for PsA include nonsteroidal anti-inflammatory drugs, corticosteroids, or conventional synthetic disease-modifying antirheumatic drugs (csDMARDs), such as methotrexate, with biologic DMARDs (tumor necrosis factor inhibitors (TNFi); interleukin (IL) 12/23 inhibitors; IL-17 inhibitors), or targeted synthetic DMARDs (such as apremilast) recommended in patients with an inadequate response to csDMARDs [2, 3]. However, not all patients achieve satisfactory disease control. In patients with rheumatic disease (PsA, rheumatoid arthritis (RA), psoriasis, or ankylosing spondylitis) receiving TNFi therapy for up to 1 year (etanercept, adalimumab, or infliximab), persistence was 42 – 56%, with 12 – 25% of patients restarting after a treatment gap and 12 – 13% switching therapies [4]. This would suggest that there is a significant unmet need for new therapies with novel mechanisms of action for patients with PsA. 

Tofacitinib is an oral Janus kinase inhibitor for the treatment of PsA [5, 6, 7]. The efficacy and safety of tofacitinib 5 and 10 mg twice daily in combination with a csDMARD have been demonstrated in two phase 3 trials of up to 12 months’ duration in patients with active PsA and an inadequate response to csDMARD or TNFi therapy (OPAL Broaden (NCT01877668) [5]; OPAL Beyond (NCT01882439) [6]), and is being investigated in an ongoing long-term extension study (OPAL Balance (NCT01976364)). 

Tofacitinib is also approved for the treatment of patients with moderately to severely active RA [7]. Detailed population pharmacokinetics (PK) characteristics of tofacitinib in healthy subjects and in patients with RA were presented in the clinical pharmacology and biopharmaceutics review published by the Center for Drug Evaluation and Research (US Food and Drug Administration; FDA) as part of the Summary Basis of Approval [8]. In brief, the PK of tofacitinib is characterized by rapid absorption and elimination, with a time to peak concentration of ~ 0.5 – 1 hour and a half-life of ~ 3 hours. Steady-state PK is predictable from single-dose data, with no evidence of unexpected systemic accumulation. In general, systemic exposure of tofacitinib increases with dose in a dose-proportional manner without regard to duration or population [8]. Dosing recommendations for patients with moderate and severe renal impairment, as described in the currently approved prescribing information for the use of tofacitinib in patients with RA or PsA [7], were derived from a separate phase 1 study that evaluated the change in exposure in subjects with normal, mild, moderate, or severely impaired renal function [9]. 

Using data from two phase 3 studies in adult patients with active PsA, the objectives of this analysis were to a) characterize the PK of tofacitinib in patients with active PsA, and b) evaluate the impact of baseline characteristics (patient-specific factors or intrinsic covariates) on tofacitinib disposition. 

## Materials and methods 

The Institutional Review Boards and/or Independent Ethics Committees approved the studies at each investigational center. Both studies included in this analysis were conducted in compliance with the Declaration of Helsinki and International Conference on Harmonisation Good Clinical Practice Guidelines [5, 6]. All patients provided written, informed consent. 

### Study design and assessments 

Key study design features and sampling schemes from the two phase 3 studies (OPAL Broaden, OPAL Beyond) used for this PK analysis are provided in Table 1 [5, 6]. The PK dataset consisted of 3,252 tofacitinib plasma concentration measurements from 650 tofacitinib-treated patients. Tofacitinib concentrations in plasma were measured using a previously described, fully validated, quantitative, high-performance liquid chromatography-tandem mass spectrometry assay [10]. 

### Data analysis 

The population PK analysis was conducted using a nonlinear mixed-effects modeling approach using the NONMEM version 7.3 software package (ICON Development Solutions, Hanover, MD, USA), with Perl-Speaks-NONMEM version 4.2.0 as supporting software. R version 3.1.2 (R Development Core Team) was used for data handling, exploratory data analysis, and creation of graphs. The estimation method was first-order conditional with interaction. The analyses were conducted using a population PK modeling approach, implemented as follows: base structural model development; random-effects model development; full model development; assessment of model predictive performance (model validation). All PK observations without recorded or missing sampling/dosing times and dates were excluded from the analysis. 

### Base model and random-effects model development 

The PK models explored were based on previous experience with RA and psoriasis patient populations and based on a prespecified analysis plan [10, 11]. The PK model based on the RA population was used as a starting point for model development. One-compartmental disposition models, parameterized in terms of apparent oral clearance (CL/F) and apparent volume of distribution (V/F) with first-order (with or without lag time) or zero-order absorption, were explored (limited samples in the absorption phase of the concentration-time profile). A one-compartment disposition model with first-order absorption and lag time was selected for this analysis. 

Intraindividual variability (IIV) of CL/F and V/F was evaluated using exponential variance models with a covariance term. A scaling parameter was used to describe the IIV of V/F on the IIV of CL/F. Residual variability (random effects) was evaluated using a proportional-error model. 

### Full model development 

Population parameters, including fixed-effects parameters (covariate coefficients and structural model parameters) and random-effects parameters, were estimated. A full covariate modeling approach was used, emphasizing parameter estimation rather than stepwise hypothesis testing. Inferences about the clinical relevance of parameters were based on the resulting parameter estimates of the full model and measures of estimation precision (asymptotic standard errors, bootstrap 95% confidence intervals (CIs)). 

Predefined covariates and covariate-parameter relationships were identified based on exploratory graphics, scientific interest, mechanistic plausibility, or prior knowledge from RA studies. Covariates evaluated in the PK model were baseline age, baseline body weight, sex, race (White, Asian, Black, other), and ethnicity (Hispanic or non-Hispanic). Baseline creatinine clearance (BCCL; calculated from the Cockcroft-Gault equation) and baseline C-reactive protein (BCRP) were evaluated as potential predictors of CL/F; age and weight were evaluated as predictors of V/F. The continuous covariates (body weight, age, creatinine clearance, and disease-related covariate BCRP) were incorporated as power functions, normalized to the baseline values (Equation 1). 


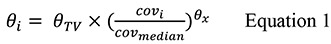


where *θ* *_i_* is the value of the parameter for the *i* *^th^* individual, *θ* *_TV_* is the typical value of the parameter of the population, *cov* *_i_* is the value for the covariate for the individual, *cov* *_median_* is the approximate median value of the covariate in the study population, and *θ* *_x_* is the effect of the covariate on the parameter. 

Each categorical covariate (sex, race, and ethnicity) was entered into the model as shown below (Equations 2 and 3). 


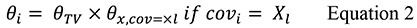



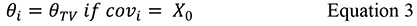


Where *θ* *_x,cov=Xl_* is the effect of the covariate belonging to the category *l*, where *l* goes from 0 (reference category) to *m* (the number of categories – 1). 

The impact of intrinsic patient characteristics on the area under the concentration-time curve (AUC) and peak concentration was estimated and expressed as the magnitude of change relative to the baseline parameters of a reference patient. For this analysis, a reference patient was set to be White, male, Hispanic, body weight 83.3 kg, age 50 years, BCRP 0.49 mg/dL, BCCL 120 mL/min. These parameters were primarily based on the central tendency of these covariates as well as precedence and prevailing practice. For ethnicity, Hispanic patients were used as the baseline typical patient for simulations. 

### Model evaluation 

The goodness-of-fit of different models to the data was evaluated using the following criteria: change in objective function value, visual inspection of various diagnostic plots, and precision of the parameter estimates. Visual predictive checks (VPCs) were performed for the selected PK models to qualify the models with respect to the prediction of the concentration data. 

## Results 

The study population consisted of 650 tofacitinib-treated patients (290 males, 360 females) with sufficient data for analysis. Patients ranged from 18 to 78 years of age, with body weights ranging from 38.1 to 159.7 kg (Table 2). The distribution of race was: 93.9% White, 0.5% Black, 3.1% Asian, and 2.6% “Other”. Hispanic patients and non-Hispanic patients constituted 10.6% and 89.4% of the population, respectively. 

A one-component model with first-order absorption and lag time was selected for this analysis. IIV of CL/F was modeled using exponential variance models with a covariance term. A scaling parameter was used to describe the impact of the IIV of the V/F on the IIV of CL/F. Residual random effects were described with two proportional-error models for concentrations collected > 5 hours postdose and ≤ 5 hours postdose. The base model adequately described the data. 

The typical estimates of CL/F and V/F from the base model were 23.5 L/h and 113 L, respectively, with relative standard errors of < 2%. The IIV estimate of CL/F was 35%. The scaling parameter used to describe the IIV of V/F on the IIV of CL/F was 0.518. Residual variability for observations with time after dose (TAD) ≤ 5 hours or TAD > 5 hours was 24% and 60%, respectively. Shrinkage estimates for CL/F from the base model were 4.24%. 

**Figure 1.**
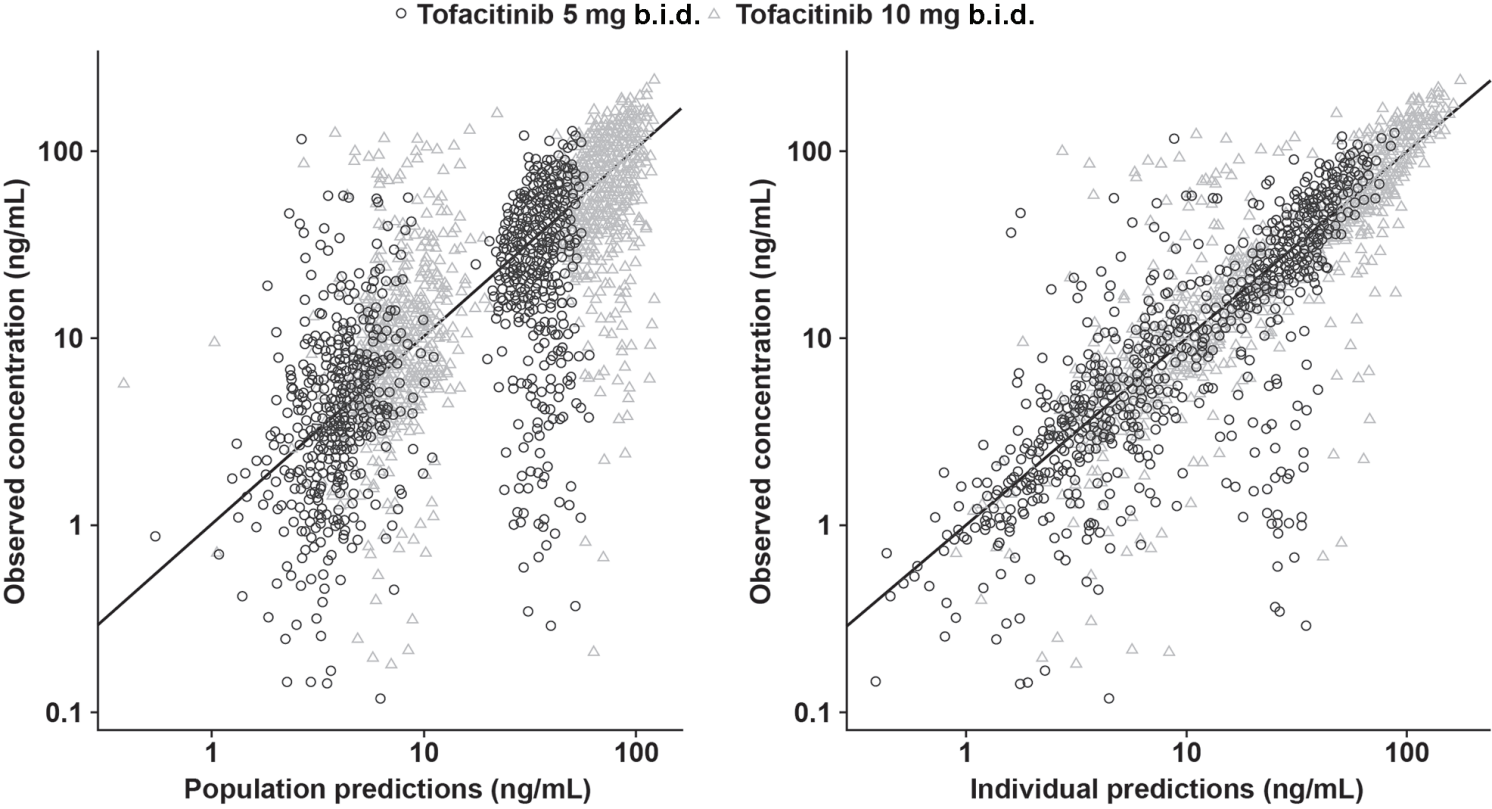
Goodness-of-fit – observed tofacitinib concentration vs. population predictions and individual predictions for the full model. Solid lines in the left panel represent the reference line for identity; solid lines in the right panel represent zero conditional weighted residual. b.i.d. = twice daily.

**Figure 2.**
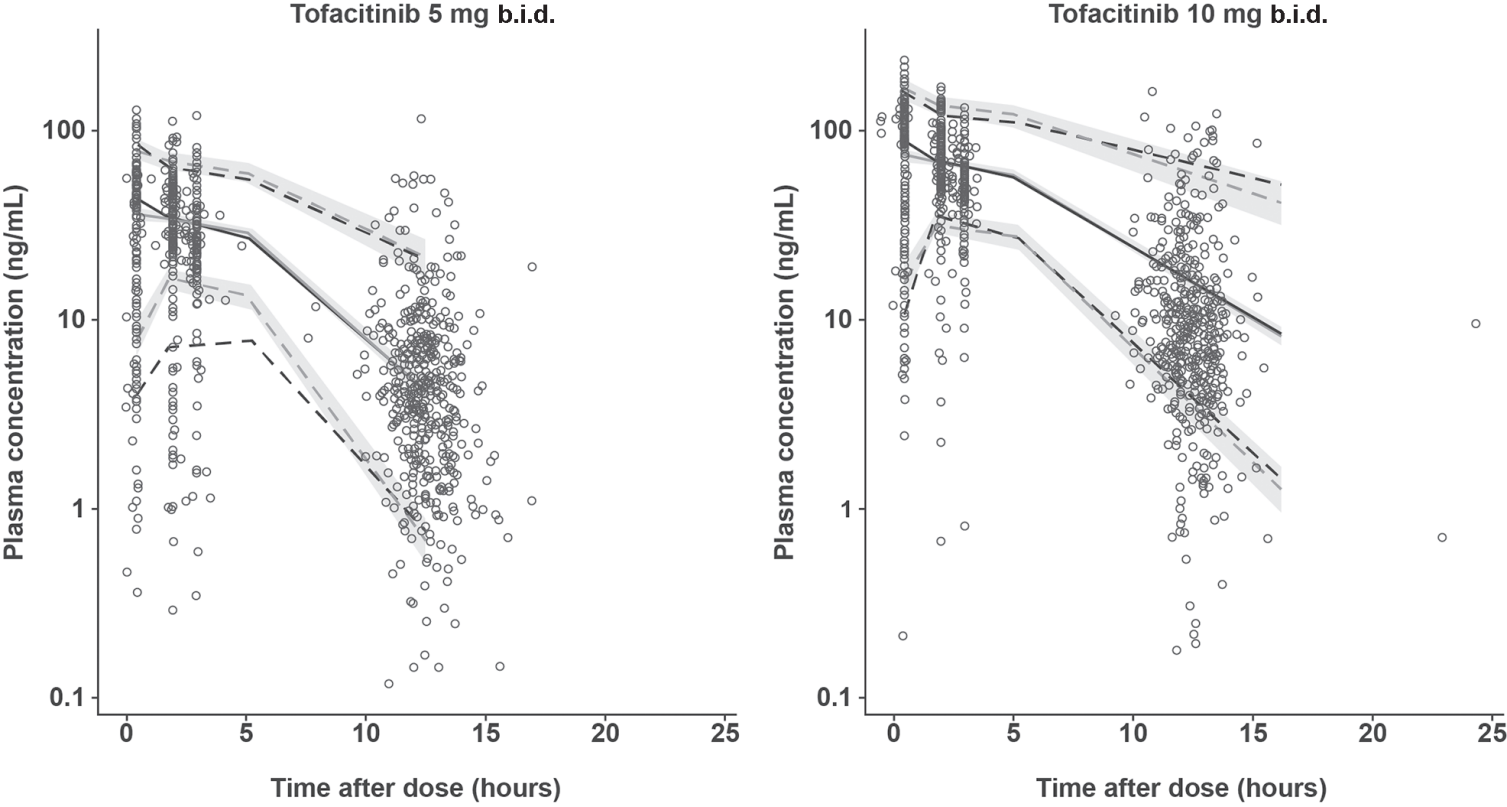
Visual predictive check stratified by dose group for the full model. Black dashed lines present 90% CI (95% upper limit and 5% lower limit) of observed data. Black solid line is median (50%) of observed data. Grey dashed lines present 90% predictive interval (95% upper limit and 5% lower limit) based on simulations. Grey solid line presents median based on simulations. Grey shaded area is predicted 95% CI of upper limit, lower limit, or median (50%) based on simulations. b.i.d. = twice daily; CI = confidence interval.

The predefined covariates were added to CL/F and V/F in the base structural model to create a full covariate model. The effect of body weight on CL/F was fixed to zero, as initial runs estimated a biologically implausible, negative exponent for the same. The parameter estimates and model fit for models with body weight effect estimated or fixed to zero were very similar. The final full model resulted in improved goodness-of-fit compared with the base model, an improvement in objective function value of ~ 333 points, having added 11 estimable parameters (Figure 1). Model evaluation using VPCs revealed that the full model provided a reliable description of the data with good precision for structural model and variance parameter estimates (Figure 2). Analysis of the full model, stratified by study, was also validated using VPCs (Supplemental Figure 1). 

The parameter estimates for the full covariate model and bootstrap results are presented in Table 3. Based on the covariate model, the typical estimates (95% CI) of PK model parameters of the reference (or typical) individual were 20.4 (18.6, 21.8) L/h, 110 (108, 113) L, and 13.8 (12.1, 16.6) L/h, for CL/F, V/F, and absorption rate constant, respectively. 

The impact of these covariates on systemic exposure was evaluated by generating point estimates using the individual parameters from the population PK. An elderly patient (80 years of age) was estimated to have 8.97% lower CL/F compared with the CL/F of a 50-year-old patient. Similarly, the V/F was estimated to be 9.9% lower for an 80-year-old patient vs. a 50-year-old patient. Females were estimated to have 5.4% higher typical CL/F value compared with males, and typical CL/F in non-Hispanic patients was 12.3% higher than in reference Hispanic patients. A typical patient with a BCCL of 50 mL/min had an estimated reduction in CL/F of 24.3% compared with a patient with BCCL 120 mL/min (median value in the analysis dataset), and a patient with a BCCL of 60 mL/min (minimal value designated as mild renal impairment, according to FDA guidance [12]) had an estimated reduction in CL/F of 19.8% compared with a patient with BCCL 120 mL/min. A patient with BCRP of 3.0 mg/dL was predicted to have a 3.6% lower CL/F vs. a typical patient with BCRP of 0.49 mg/dL. Typical V/F estimates for patients weighing 61 or 109 kg (10^th^ and 90^th^ percentile of body weight, respectively) were ~ 19% lower or 20% higher compared with patients with a body weight of 83.3 kg, respectively. 

**Figure 3.**
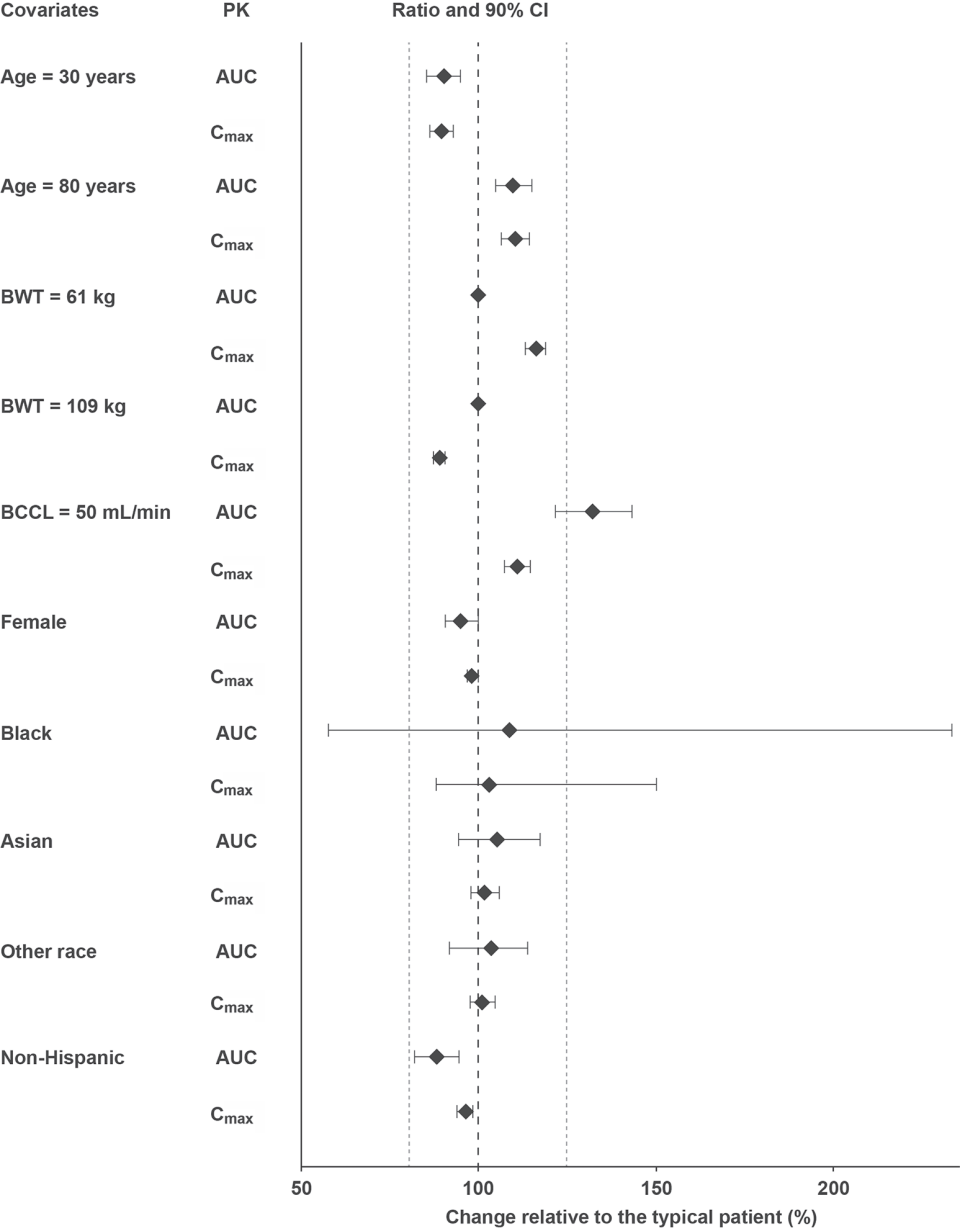
Impact of covariates on the PK of tofacitinib in patients with psoriatic arthritis. Grey dotted line represents limits of a range from 80 to 125%. Magnitude of change is presented in reference to a typical baseline patient (White, male, Hispanic, body weight 83.3 kg, age 50 years, BCRP 0.49 mg/dL, BCCL 120 mL/min). Weights of 61 and 109 kg represent the 10^th^ and 90^th^ percentiles of body weight. BCCL of 50 mL/min with reference to the typical patient (49 mL/min was the lowest BCCL in the analysis). Point estimates for AUC and C_max_ were generated using the individual parameters obtained using the population PK model, and the CIs were generated from 1,000 nonparametric bootstrap runs. AUC = area under the concentration-time curve over a dosing interval; BCCL = baseline creatinine clearance; BCRP = baseline C-reactive protein; BWT = baseline body weight; CI = confidence interval; C_max_ = maximum steady-state concentration; PK = pharmacokinetics.

The impact of covariate effects (magnitude of change (90% CI)) on secondary parameters is demonstrated on the reference population in Figure 3. The point estimates of AUC and maximum concentration (C_max_) shown were generated using the individual parameters obtained using the population PK model, and the CIs were generated from the 1,000 nonparametric bootstrap runs. With the exception of BCCL, point estimates of the AUC at steady state and C_max_ change relative to typical baseline patient values ranged from 88 to 110%, and from 89 to 116%, respectively. For a patient with a BCCL of 50 mL/min, the AUC at steady state was estimated to be 32% higher relative to a reference patient with a BCCL of 120 mL/min. There were no patients with BCCL values below 49 mL/min in the analysis dataset. The point estimates of the AUC and C_max_ ratios, and the associated 90% CI, excluded ≥ 19% difference (except patients of Black race), indicating no major differences in tofacitinib exposure over the range of ages and body weights studied, as well as race, ethnicity, and sex. As there were only 3 patients of Black race in this dataset, the respective CIs were large. 

## Discussion 

The characterization of the PK of an agent in the intended disease population is an important objective in drug development, with clear guidelines for its effective assessment in a clinical setting provided by the FDA [13]. Identifying patient characteristics that could alter the PK profile of an agent in a clinically meaningful way informs its optimal use in that specific therapeutic setting. 

This study, which used data from two global phase 3 trials (OPAL Broaden and OPAL Beyond) [5, 6] in adult patients with active PsA, aimed to describe the PK of tofacitinib in this population and to evaluate the effects of covariates on the variability in PK parameter estimates. A one-compartment model, based on a tofacitinib PK model in a patient population with RA with first-order absorption and an absorption lag, was found to adequately describe the population PK of tofacitinib in patients with active PsA and allowed the evaluation of the patient-specific covariates on the systemic concentrations in these patients. 

The results of this PK analysis demonstrate that the typical population estimate of tofacitinib CL/F in the reference patients with active PsA (20.4 L/h) was similar to such estimates in patients with RA (18.4 L/h) [8] and psoriasis (26.7 L/h) [11], but lower compared with the clearance estimates from a pooled analysis of healthy subjects (34.9 L/h) [8]. Tofacitinib is primarily (~ 70%) metabolized hepatically [10, 14, 15], with the CYP450 enzymes CYP3A4 and CYP2C19 believed to be responsible for ~ 55% and ~ 15% of tofacitinib’s clearance in healthy subjects, respectively [10, 15]. One explanation for this lower CL/F in patients with immune-mediated inflammatory diseases relative to healthy subjects is believed to be the reported downregulation of CYP450 by inflammatory stimuli [16, 17]. This would confirm the substantial inflammation burden of patients with PsA, psoriasis, and RA compared with healthy subjects. 

This analysis in patients with PsA has shown that IIV of CL/F was comparable with that previously observed in patients with RA and psoriasis (IIV of CL/F in PsA was 32%, compared with 27% in RA [8] and 28% in psoriasis [11]). Consistent with previous studies of RA and psoriasis, the only factor that led to clinically relevant changes in tofacitinib AUC in this analysis was BCCL (Figure 3). A patient with a creatinine clearance of 50 mL/min (lowest value in this dataset was 49 mL/min) was estimated to have 24.3% lower CL/F compared with a patient with a creatinine clearance of 120 mL/min (median value in this analysis dataset). In a similar comparison, a patient with a creatinine clearance of 60 mL/min (minimal value, designating mild impairment) was estimated to have a 19.8% lower CL/F. These results were consistent with the known contribution of renal excretion (~ 30%) to the total elimination of tofacitinib [10, 14]. These results were also consistent with regression analysis of pooled data from phase 1 renal impairment studies [8], which were the basis for dose adjustment recommendations in the currently approved prescribing information for the use of tofacitinib in patients with RA or PsA with moderate and severe renal impairment [7]. There were only 3 patients of Black race in this dataset, which resulted in large CIs. However, population PK evaluations in patients with RA that included 19 patients of Black race showed ≤ 5% difference (CIs excluded ≥ 25% difference) in AUC and C_max_ relative to patients of White race [8]. In addition, no clinically relevant differences based on race were observed in patients with psoriasis [11]. Taken together, the data suggest that no major differences in tofacitinib exposure are expected in patients of Black race with PsA compared with patients of White race. 

Based on this population PK evaluation in patients with active PsA, tofacitinib does not require dose modification or restrictions for age, body weight, gender, race, ethnicity, or baseline disease severity in adult patients with active PsA, similar to observations previously reported in patients with RA [8]. 

## Conclusion 

Overall, this study provides an in-depth characterization of the PK of tofacitinib in patients with active PsA and provides a quantitative rationale for dose-modification recommendations of tofacitinib based on intrinsic characteristics in this patient population. Tofacitinib did not require dose modification or restrictions for age, body weight, sex, race, ethnicity, or baseline disease severity in patients with active PsA. Furthermore, the relationship between tofacitinib CL/F and BCCL was found to be consistent with the known contribution of renal excretion to the total apparent clearance of tofacitinib. 

## Acknowledgment 

The authors thank the patients who participated in the OPAL Broaden and OPAL Beyond clinical studies, and acknowledge the data preparation work by the Specialized Data Professional Team, led by Luai Alzoubi. 

Medical writing support, under the guidance of the authors, was provided by Richard Knight, PhD, at CMC Connect, a division of McCann Health Medical Communications Ltd, Macclesfield, UK, and was funded by Pfizer Inc, New York, NY, USA, in accordance with Good Publication Practice (GPP3) guidelines (Ann Intern Med. 2015; *163:* 461-464). 

## Data-sharing statement 

Upon request, and subject to certain criteria, conditions, and exceptions (see https://www.pfizer.com/science/clinical-trials/trial-data-and-results for more information), Pfizer will provide access to individual de-identified participant data from Pfizer-sponsored global interventional clinical studies conducted for medicines, vaccines, and medical devices (1) for indications that have been approved in the US and/or EU or (2) in programs that have been terminated (i.e., development for all indications has been discontinued). Pfizer will also consider requests for the protocol, data dictionary, and statistical analysis plan. Data may be requested from Pfizer trials 24 months after study completion. The de-identified participant data will be made available to researchers whose proposals meet the research criteria and other conditions, and for which an exception does not apply, via a secure portal. To gain access, data requestors must enter into a data access agreement with Pfizer. 

## Funding 

This study was sponsored by Pfizer Inc. 

## Conflict of interest 

R. Xie, Q. Wang, K. Kanik, T. Nicholas, and S. Menon are shareholders and employees of Pfizer Inc. C. Deng is a shareholder and was an employee of Pfizer Inc during the time of this analysis. 

**Table 1.** Overview of tofacitinib phase 3 studies in patients with PsA included in the population PK analysis.

Study identifier	Design/total duration	Tofacitinib treatment groups	Number of patients in dataset	Sampling schedule
OPAL Broaden [5] (NCT01877668)	Phase 3, randomized, double-dummy, double-blind, placebo-controlled and active-controlled (adalimumab 40 mg Q2W), parallel-group study in csDMARD-IR (and TNFi-naïve) patients with active PsA, receiving a background csDMARD Total duration: 12 months	Tofacitinib 5 mg b.i.d.	104	Month 1 ± 3 days^a^. Predose^b^ and 2 hours after in-clinic dose Month 4 ± 7 days. Predose^b^, 0.5, 2, and 3 hours after in-clinic dose Month 6 ± 7 days^c^. Predose^b^, 0.5, 2, and 3 hours after in-clinic dose
		Tofacitinib 10 mg b.i.d.	104	
		Placebo → tofacitinib 5 mg b.i.d. at month 3	52	
		Placebo → tofacitinib 10 mg b.i.d. at month 3	50	
OPAL Beyond [6] (NCT01882439)	Phase 3, randomized, double-blind, placebo-controlled, parallel-group study in TNFi-IR patients with active PsA, receiving a background csDMARD Total duration: 6 months	Tofacitinib 5 mg b.i.d.	127	Month 1 ± 3 days^a^. Predose^b^ and 2 hours after in-clinic dose Month 4 ± 7 days. Predose^b^, 0.5, 2, and 3 hours after in-clinic dose Month 6 ± 7 days^c^. Predose^b^, 0.5, 2, and 3 hours after in-clinic dose
		Tofacitinib 10 mg b.i.d.	126	
		Placebo → tofacitinib 5 mg b.i.d. at month 3	64	
		Placebo → tofacitinib 10 mg b.i.d. at month 3	60	

^a^PK samples were only taken for patients receiving either tofacitinib 5 or 10 mg b.i.d. at month 1. ^b^Predose sampling occurred 12 ± 2 hours after the evening dose of study medication was taken and immediately prior to the in-clinic dose of study medication. ^c^PK samples were taken at month 6 for those patients who did not have PK samples at month 4. b.i.d. = twice daily; csDMARD = conventional synthetic disease-modifying antirheumatic drug; IR = inadequate responder; PK = pharmacokinetics; PsA = psoriatic arthritis; Q2W = every 2 weeks; TNFi = tumor necrosis factor inhibitor.

**Table 2.** Baseline demographics and characteristics of the study population.

	Total patient population (n = 650)
Continuous variables, mean (SD) [range]	
Age, years	49.0 (12.1) [18 – 78]
BWT, kg	84.8 (19.0) [38.1 – 159.7]
BCCL, mL/min	123.4 (37.7) [49.1 – 348.5]
BCRP, mg/dL	1.1 (2.0) [0.0 – 16.4]
Categorical variables, n (%)	
Female	360 (55.4)
Race	
White	610 (93.9)
Black	3 (0.5)
Asian	20 (3.1)
Other	17 (2.6)
Ethnicity	
Hispanic	69 (10.6)
Non-Hispanic	581 (89.4)

BCCL = baseline creatinine clearance; BCRP = baseline C-reactive protein; BWT = baseline body weight; SD = standard deviation.

**Table 3.** Parameter and covariate parameter estimates for the full population PK model.

Parameter estimates and bootstrap results				
	Full model		Bootstrap^a^	
	Estimates (RSE %)	IIV (RSE %)	Median (IIV %)	95% CI (95% CI for IIV)
CL/F, L/h	20.4 (4.7)	31.7 (3.7)	20.2 (31.3)	18.6, 21.8 (28.2, 34.8)
V/F, L	110 (1.2)	–	110	108, 113
Ka, /h	13.8 (7.9)	198 (4.2)	14.0 (198)	12.1, 16.6 (180, 222)
Proportional error, TAD ≤ 5 hours, %	22.9 (3.8)	65.8 (5.5)	22.8 (66.4)	21.0, 24.7 (57.2, 73.1)
Proportional error, TAD > 5 hours, %	52.6 (4.7)	65.8 (5.5)	52.5 (66.4)	48.0, 57.3 (57.2, 73.1)
Lag time, h	0.3 (0.8)	–	0.3	0.3, 0.3
Scaling parameter	0.5 (9.0)	–	0.5	0.4, 0.5
**Covariate parameter estimates**				
**PK parameter**	**Covariate**	**Estimate**	**%RSE**	**95% CI^a^**
CL/F, L/h	Age	–0.20	–28.6	–0.31, –0.08
CL/F^b^, L/h	BWT	0 (FIX)	NA	0, 0 (FIX)
CL/F, L/h	BCCL	0.32	16.7	0.21, 0.42
CL/F, L/h	BCRP	–0.02	–46.0	–0.04, 0.00
CL/F, L/h	Black	0.91	54.9	0.42, 1.78
CL/F, L/h	Asian	0.95	6.07	0.83, 1.08
CL/F, L/h	Other race	0.96	5.91	0.86, 1.12
CL/F, L/h	Non-Hispanic	1.12	4.75	1.04, 1.24
CL/F, L/h	Female	1.05	2.77	0.99, 1.11
V/F, L	Age	–0.22	–22.6	–0.32, –0.13
V/F, L	BWT	0.68	8.37	0.55, 0.79

^a^760 in 1,000 runs minimized successfully. ^b^Effect of BWT on CL/F was fixed to 0, and RSE and CI could not be calculated. Reference patient defined as: White, male, Hispanic, body weight 83.3 kg, age 50 years, BCRP 0.49 mg/dL, BCCL 120 mL/min. BCCL = baseline creatinine clearance; BCRP = baseline C-reactive protein; BWT = baseline body weight; CI = confidence interval; CL/F = apparent oral clearance; IIV = interindividual variability; Ka = first-order absorption rate; NA = not available; PK = pharmacokinetics; RSE = relative standard error; TAD = time after dose; V/F = apparent volume.

## Supplemental material

Supplemental material Supplementary Figure
