# Supplementary material for: Population pharmacokinetics of tofacitinib in patients with psoriatic arthritis
Source: Int J Clin Pharmacol Ther. 2019 Jul 19;57(9):464–73. doi: 10.5414/CP203516 (PMC6704728; doi:10.5414/CP203516)
Supplement: Supplemental material [file intjclinpharmacol-57-464-S01.pdf]

Supplement

Supplementary Figure 1. Visual predictive check stratified by study for the full model.

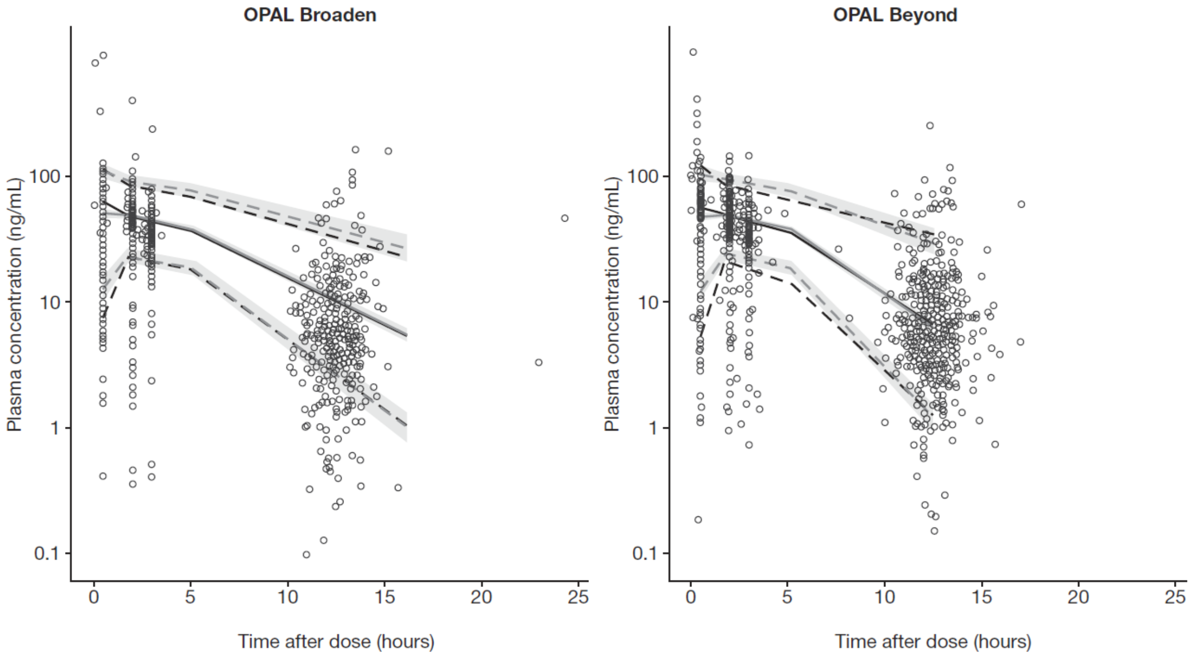

Black dashed lines present 90% CI (95% upper limit and 5% lower limit) of observed data. Black solid line is median (50%) of observed data. Grey dashed lines present 90% predictive interval (95% upper limit and 5% lower limit) based on simulations. Grey solid line presents median based on simulations. Grey shaded area is predicted 95% CI of upper limit, lower limit, or median (50%) based on simulations.

CI = confidence interval.
